# Endogenized polinton-like viruses in the dinoflagellate Oxyrrhis marina uncover novel PolB fusion

**DOI:** 10.1099/jgv.0.002200

**Published:** 2025-12-22

**Authors:** Ronie Haro, Lucie Gallot-Lavallée, John M. Archibald, Claudio H. Slamovits

**Affiliations:** 1 Department of Biochemistry & Molecular Biology, Dalhousie University, Halifax, NS, B3H 4R2, Canada; 2 Institute for Comparative Genomics, Dalhousie University, Halifax, NS, B3H 4R2, Canada

**Keywords:** endogenous, polinton, polymerase B, protists, viruses

## Abstract

Marine viruses are ubiquitous entities that impact the biology of a large fraction of prokaryotic and eukaryotic diversity. Dinoflagellates are heterotrophic, mixotrophic and photosynthetic eukaryotes known for their large and complex nuclear genomes, permanently condensed chromosomes and ability to form toxic algal blooms. We used long-read sequencing to explore the genome of the dinoflagellate *Oxyrrhis marina* and discovered a novel, endogenized ~25 kbp polinton-like virus (PLV). *O. marina* PLV (OmPLV) resides primarily in low GC content regions and exhibits a distinctive codon usage pattern, suggesting recent endogenization. OmPLV fragmentation was also observed, suggesting that this element is evolving towards a transposable element-like lifestyle. Notably, OmPLV encodes a unique ~1,500 amino acid fused DNA Helicase SF1–pPolB, representing the largest replication proteins reported so far in Preplasmiviricota. Phylogenetic analysis of the major capsid protein positions OmPLV as a novel PLV lineage related to giant viruses. The discovery of OmPLV provides crucial insight into the evolution and diversification of dsDNA viruses and serves as an important reference point for elucidating the role of endogenized viruses in expansion of dinoflagellate genomes.

## Data Summary

Sequences reported in this study were deposited in GenBank under accession number PX556662. All host-viral contigs, viral genomes, proteins, protein secondary structure predictions and alignments are available from the Zenodo data repository DOI 10.5281/zenodo.14962255.

## Introduction

The non-coding fraction of eukaryotic nuclear genomes predominantly comprises transposable elements (TEs) and endogenous viral elements, whose boundaries are fluid and overlapping [1]. Preplasmiviricota are emergent dsDNA viruses, endogenized versions of which have been identified in the genomes of six of the seven major eukaryotic lineages [2]. Their genomes range from 15 to 40 kbp in size and are classified into four main categories: virophages, polintons/mavericks, polinton-like viruses (PLVs) and adenoviruses [3]. The viral capsid of Preplasmiviricota is composed of double jelly-roll (DJR) proteins that assemble into icosahedral virus particles [4,5]. These viruses typically have a highly reduced gene set, including a DNA-packaging ATPase and maturation protease involved in capsid morphogenesis. Additionally, they encode a protein-primed DNA polymerase B (pPolB) and retrovirus-derived integrase (RVE-INT), which are essential for viral genome replication and integration, respectively. However, the gene content can vary significantly among lineages of small dsDNA viruses [3].

The coding capacity of Preplasmiviricota is dynamic, with gene replacements, losses and fusions all having been described. For instance, while integrase and pPolB are often absent in PLVs, tyrosine recombinase (Y-rec) and GIY-YIG endonuclease are usually present and likely play roles in viral integration [6,7]. Genes typically found in large dsDNA *Nucleocytoviricota* viruses (NCLDVs) are also found in some polintons (e.g. resolvase, Bro-N and HNH endonuclease) [8,9]. These shared genes may be the product of recombination or lateral gene transfer events, hinting at a convoluted dsDNA virus diversification [10]. Fused genes involved in viral genome replication have been discovered in PLVs, such as the Tlr1-helicase in the ciliate *Tetrahymena thermophila* Tlr1-PLV, which features a truncated pPolB fused to a helicase (SF1H) and a GIY-YIG endonuclease domain [11]. Similar fusions, including helicase superfamily 3 (SF3H) with DNA polymerase A (PolA-SF3H) or archaeo-eukaryotic primase-polymerases (AEP-SF3H), are also observed in related PLVs [6,7].

Analysis of evolutionarily conserved replication enzymes like pPolB has proven useful for elucidating the origin of dsDNA viruses, establishing a connection between Preplasmiviricota and eukaryotic non-viral elements, such as mitochondrial and cytoplasmic plasmids [12]. For example, phylogenetic analysis of pPolB suggests that mitochondrial plasmids and polintons likely evolved from prokaryotic tectiviruses [8]. Additionally, adenoviruses, NCLDVs and virophages are thought to have emerged from an ancestral polinton-like element that escaped the nucleus of early eukaryotes – the so-called ‘nuclear escape hypothesis’. This evolutionary scenario is further supported by the recent discovery of two pPolB N-terminal domains: the terminal protein (TP) and viral ovarian tumour-like cysteine deubiquitinase (vOTU), likely involved in the protein-primed replication initiation and the cleavage of TP from the pPolB polypeptide, respectively [4]. The presence or absence of these domains, along with the active replacement of domains in pPolB and other DNA polymerases, appears correlated with the origin and diversification of Preplasmiviricota [4]. Our understanding of the diversity of Preplasmiviricota nevertheless remains limited; its gene repertoire is still expanding, and key viral elements are yet to be discovered.

Dinoflagellates are among the most abundant protists in the world’s oceans. As primary producers, they can form large and toxic blooms, commonly known as red tides. Viral infection may contribute to the decline of these blooms through the ‘viral shunt’ process, whereby algal biomass is transformed into dissolved organic matter [13]. Dinoflagellates are known for possessing unique nuclear organization, including permanently condensed chromosomes, closed mitosis and expanded genomes (>200 Gbp in some cases [14]). Due to their massive genome and substantial repeat content (up to ~65% of the total genome [15]), much of the organization and coding capacity of dinoflagellate genomes remains unexplored. Consequently, evidence for endogenized dsDNA viruses in dinoflagellates is limited to individual genes such as giant virus HcDNAV-like PolB and HNH endonuclease, identified in the bloom-forming species *Heterocapsa circularisquama* [16,17]. Recently, long-read sequence-based genome assemblies revealed ~2,500 polinton and polinton-like viral elements in the genome of the cold-adapted dinoflagellate *Polarella glacialis* [18], making it one of the most heavily ‘infected’ protist genomes ever reported. Therefore, dinoflagellate genomes may represent an untapped reservoir of endogenous viral diversity.

In this study, we surveyed the nuclear genome of the free-living dinoflagellate *Oxyrrhis marina* to investigate viral elements. We identified a novel endogenized ~25 kbp PLV, herein referred to as *O. marina* PLV (OmPLV). This PLV appears to represent an undescribed viral lineage with some affinity to NCLDVs and Mriyaviruses (small relatives of giant viruses [19]). Additionally, we discovered a novel fusion of Helicase SF1H with a complete pPolB, including both exonuclease and polymerase domains, forming a large ~1,500 amino acid protein. Homologues of this fusion protein were found in metagenomic contigs from marine environments, suggesting that it may be present in a much broader set of yet-to-be-revealed viral elements in nature.

## Methods

### DNA extraction, sequencing and assembly

*O. marina* was isolated from Curaçao and maintained as a clonal culture in F/2 media supplemented with cholesterol (8 mg ml^−1^) and Coenzyme Q_10_ (100 µg ml^−1^) [20]. DNA extraction was performed using the cetyltrimethylammonium bromide (CTAB method [21]. Pelleted cells were resuspended in prewarmed (60 °C) 2% CTAB buffer (100 mM Tris pH 8.0, 20 mM EDTA, 1.4 M NaCl, 2% Cetyl Trimethyl Ammonium Bromide and 1% Polyvinylpyrrolidone) and treated with Proteinase K (10 µg ml^−1^) for 1.5 h at 60 °C, followed by RNase A (20 µg ml^−1^) treatment for 30 min at 37 °C. DNA was purified via two rounds of phenol-chloroform-isoamyl alcohol extraction (Phe/Chl/IAA 25:24:1) and precipitated with isopropanol (12 h, RT). DNA fragments <10,000 bp in size were removed using a short-read eliminator kit (Circulomics-Pacbio).

High-molecular-weight DNA was sequenced using the PacBio Sequel II platform (Genome Quebec, Montreal), generating 9.1 million subreads with an N50 of ~15 kbp. Short-read sequencing was performed using the Illumina NovaSeq 6000 platform, producing 780 million reads. Sequence quality was evaluated with FastQC v.0.11.9, and adapters were removed using Trimmomatic v.0.39 [22]. These sequence datasets were decontaminated with Centrifuge v.1.0.4 [23], and *de novo* assembly was carried out with Flye v.2.9.2 [24], followed by two rounds of polishing using Pilon v.1.24 [25] with Illumina short reads.

### Identification and annotation of viral contigs

Candidate viral contigs were identified within the draft assembly of *O. marina* using Vibrant v.1.2.1 [26], with default settings. Contigs selected by Vibrant were at least 10 kbp in length and contained more than four ORFs beforehand predicted by Prodigal v.2.6.3 [27]. These putative viral contigs were then annotated using HMM profiles via HHsearch v.3.1 [28] using three databases: virus orthologous group [VOGDB (release 94)] (https://vogdb.org/), PFAM v.32 (2019) [29] and KEGG (March-2023 release) [30]. These regions were extracted as putative proviral elements, and their encoded peptides were analysed for viral marker genes, including major capsid protein (MCP), pPolB, ATPase and integrase, using HHpred [31] and National Center for Biotechnology Information (NCBI) conserved domain search [32] (e-value≤E-5). Viral element boundaries were defined by a drop in GC content (below) and terminal inverted repeats (TIRs), located through self-mapping analysis with Minimap2 v.2.24 [33], following Bellas *et al.* 2023. These TIRs were used to delineate the gene repertoire of the viral elements and assess their completeness. Neighbouring retrotransposons were annotated through HHsearch (e-value≤E-5) using gypsy database v.2.0 [34]. Viral genome representations were generated using the package ‘gggenes’ (https://github.com/wilkox/gggenes), and ‘gggenomes’ (https://github.com/thackl/gggenomes) implemented in R.

### GC content and codon usage

GC content variation was manually detected by inspecting the GC-skew in Geneious v.R11. A decrease in GC content relative to the host genome is a known characteristic of virophages and polintons/mavericks [18,35, 36]. Viral regions were identified and isolated based on two criteria: (1) a GC content lower than 58% found in the overall host assembly and (2) the presence of viral genes. These isolated regions were then compared to the remaining portions of viral contigs that appeared to have a host origin. GC content was calculated using SAMtools v.1.18 with a sliding window approach (300 bp window size). Statistical comparisons between viral and host regions were performed using the Wilcoxon test. Codon usage bias was assessed by comparing synonymous codon usage orderliness (SCUO) [37] statistics between viral and host ORFs. The analysis was conducted using the coRdon (https://github.com/BioinfoHR/coRdon) implemented in R. Statistical comparisons were estimated using the Wilcoxon test. The results were graphed using ‘ggplot2’ implemented in R.

### RNA-seq mapping

Previously generated *O. marina* RNA-seq data were used to estimate the expression of OmPLVs and compare them with host contigs; raw reads (SRA) (SRR1296907 and SRR1300472) were retrieved from NCBI under BioProject PRJNA231566. Trimmomatic v.0.39 [22] was used to trim the reads with a conservative setting [38]. Mapping and quantification of the reads were performed using Bowtie2 v.2.4.5 [39] and Rsem software v. 1.3.0 [40], respectively. Coverage per nucleotide was calculated with SAMtool [41], and coverage plots were created using the ‘ggplot2’ package in R.

### Phylogenetic analysis and protein structure prediction

Phylogenetic analyses of MCP and pPolB proteins were performed to infer the phylogenetic origin of OmPLV. Amino acid sequences for MCP phylogenetic reconstruction were obtained from [6,42,44]. Sequences from [6,8] were used for pPolB. These studies and datasets were selected to maintain consistency with well-characterized viral groups and to assess the potential affiliation of OmPLV. Amino acid sequences were aligned using MAFFT v.7.471 [45] and trimmed with trimAI v.1.4 [46]. Maximum likelihood (ML) phylogenetic trees were constructed using IQ-TREE [47] with 1,000 ultrafast bootstrap replicates. Best substitution models were estimated by ModelFinder [48]. Phylogenetic trees were edited using ggtreeExtra [49] (https://github.com/YuLab-SMU/ggtreeExtra) implemented in R.

The secondary structures of MCP, pPolB and the fused Helicase SF1–pPolB proteins were predicted and modelled using AlphaFold3 [50]. The amino acid sequence for MCP of the *T. thermophila* Tlr1 viral element was retrieved from NCBI (AAL73464.1). The complete fused Helicase SF1–pPolB sequence for OmPLV was assembled by aligning its amino acids using MAFFT v.7.471. The conserved motifs for the Helicase and Pol domains were identified based on previous consensus and descriptions.

## Results

### *O. marina* harbours a novel dsDNA virus

**Fig. 1.**
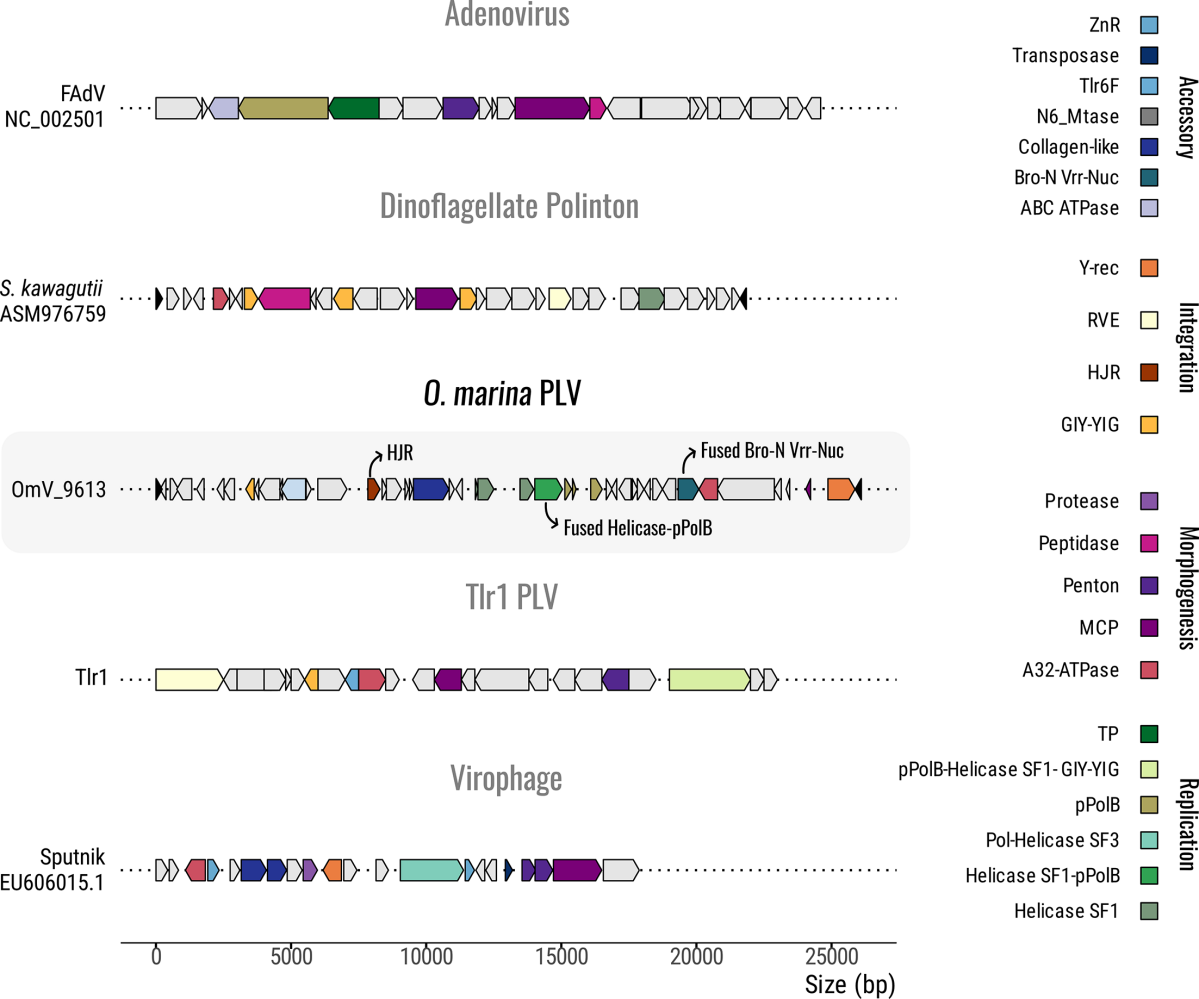
Genomic architecture of representative dsDNA viruses from the PLV supergroup. Homologous genes are colour-coded and organized into putative functional modules and accessory genes. Blocks of arrows depict viral gene content and transcriptional orientation. White-filled arrows indicate poorly conserved genes that encode unknown proteins. Prominent features of OmPLV include novel fused protein domains (Bro-N–VRR-NUC, Helicase SF1–pPolB) and the presence of HJR. Representative viral genomes were retrieved from NCBI, including frog adenovirus FAdV (NC_002501) and *Cafeteria* Sputnik virophage (EU606015.1). The Tlr1 *T. thermophila* viral element was reconstructed based on partial sequences (AF451863.1, AF45860.1) and adapted from Koonin and Krupovic [12]. The representative dinoflagellate polinton was taken from the genome assembly of *S. kawagutii* (ASM976759) contig VSDK01003953.1 (coordinates 97,440–118,633).

We used PacBio long-read sequencing to perform partial nuclear genome sequencing and *de novo* assembly of the dinoflagellate *O. marina*. N50 values for the sequenced reads and assembly were ~15 and 27 kbp, respectively. Contiguity was limited, as expected for a dinoflagellate genome, but the resulting assembly was sufficient for the purposes of characterizing repeat regions and viral content. A systematic search for endogenized viruses revealed the presence of several viral gene markers, including ATPase, helicase and pPolB. Further examination also identified an MCP, confirming the viral origin of these elements. A total of 28 endogenized viral elements were identified in our *O. marina* genomic assembly (Fig. S1, available in the online Supplementary Material; see below), all corresponding to the same viral entity (or variants thereof with highly similar or identical amino acid sequence for MCPs), but with substantial diversity in gene order and content. Four OmPLVs (9613; 16701; 12314; 10233) might remain functional with intact core genes (i.e. ATPase, MCP, Y-rec and fused Helicase-polB). These viral-derived sequences are distinct from other small dsDNA viruses, such as polintons, PLVs, adenoviruses and virophages, in that they contain uncommon and unique genes such as Holliday junction resolvase (HJR) and fused Helicase–pPolB (Fig. 1).

### OmPLV is endogenized and transcriptionally silent

**Fig. 2.**
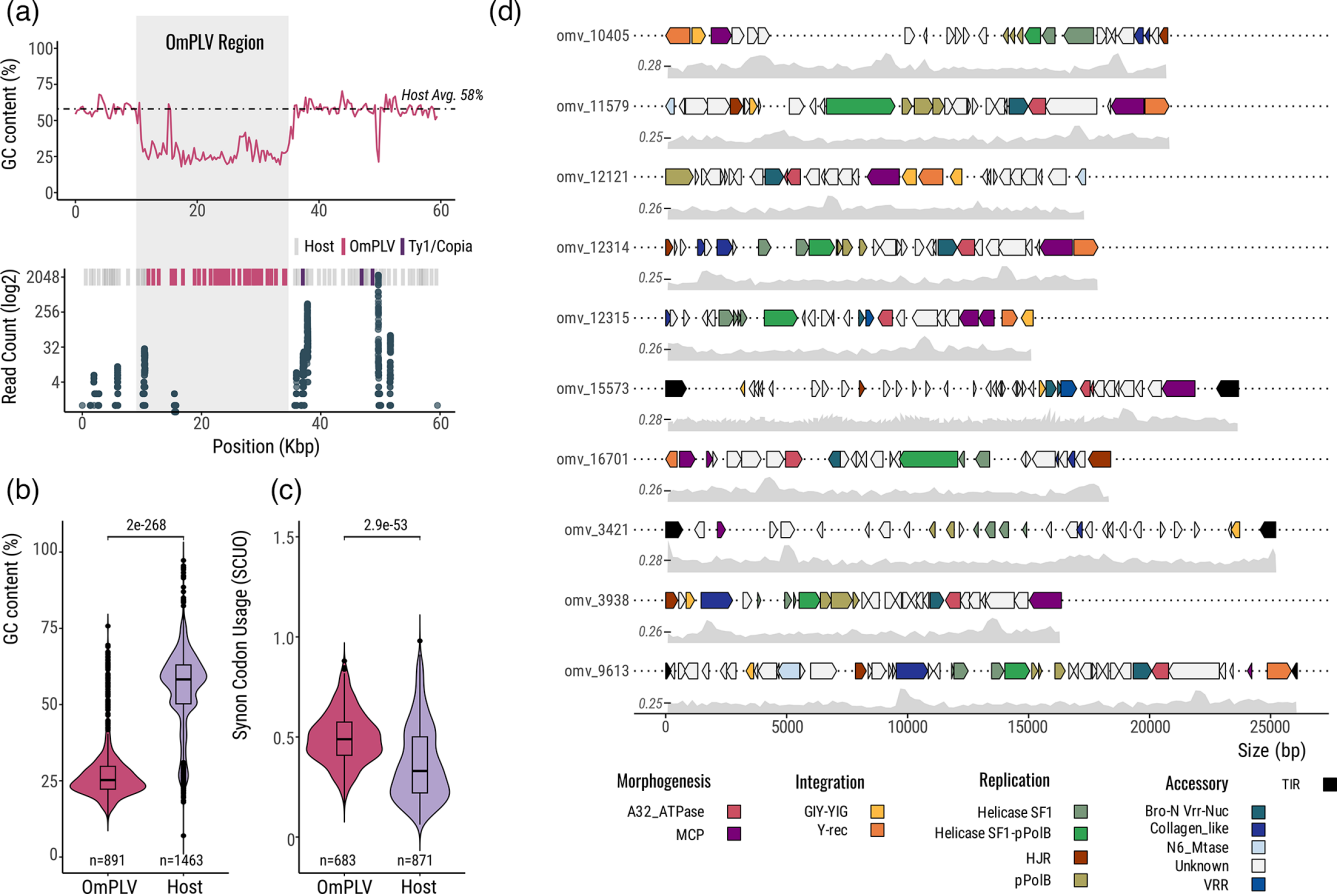
Endogenization and gene repertoire of *O. marina* OmPLV. (a) Top, Illustrative example of GC content deviation between OmPLV (grey shadow) and flanking host regions. The black dotted line denotes the average GC content for *O. marina* (58%), and the red skew indicates the fluctuation of GC along the contig. Bottom, expression profile of the host contig. Mapped RNA reads are represented by blue dots (*y*-axis). The ribbons at the top represent predicted ORFs for the host (grey and blue) and virus (red). Additional information can be found in Figs S1 and S2. (b) Comprehensive comparison of GC content (300 bp window) between OmPLV (892 windows) and the host *O. marina* (1,464 windows). (c) Codon usage bias (SCUO) comparison of OmPLV ORFs (*n*=684) and the host (*n*=872). (d) Physical map of the ten largest OmPLV genomes. Functional assignments are colour-coded according to the legend. GC content fluctuation is displayed below each element, with the average indicated on the left. Gene legend abbreviations: Helicase SF1 (superfamily 1, PF05970); pPolB, protein-primed DNA polymerase type B (PF03175); HJR (PF04848); A32_ATPase, DNA packing ATPase (PF04665); GIY-YIG endonuclease (PF01541); VRR-NUC endonuclease domain (PF08774); N6_MTase, N6-methyltransferase (PF02384); collagen-like protein (PF01391); Bro-N domain (PF02498); Y-rec, tyrosine recombinase. TIRs are depicted in black and often delineate the hypothetical full-length viral genomes. Additional OmPLVs can be found in Fig. S3. Viral genomic plots were generated, as shown in Fig. 1, and GC content was estimated using SAMtools v.1.20 and BEDtools v.2.31.0.

The 28 viral elements were identified by searching *O. marina* genomic contigs for hallmarks of recent virus endogenization such as GC content and codon usage variation between host and candidate viral genes. These genes were found to be clustered, not randomly dispersed amongst host genes, consistent with endogenization of larger viral elements [35,51,55]. OmPLV sequences are ~5 to 27 kbp in length (~16 kbp average) with conspicuously low GC content (Fig. 2a), and the contigs are sufficiently long (ranging from 2 to 92 kbp, averaging ~40 kbp) to fully capture viral endogenizations (Fig. S1). Contig depth of sequence coverage ranged from 5× to 254× (average ~80×), suggesting substantial copy number variation in the *O. marina* genome. Comparative analysis showed that the viral regions are ~25% GC, significantly lower than 58% for the host genome (Fig. 2b). Similarly, the codon usage (SCUO) of viral ORFs (~0.5) differs significantly from that of the host (0.3), indicating a higher codon usage bias in OmPLV genes (Fig. 2c). These data suggest recent OmPLV endogenization; given that most MCP amino acid sequences are identical between the different viral copies, this probably occurred once or a few times at most. Viral DNA integration was likely followed by intragenomic spread, landing in non-coding regions near retrotransposons.

We next searched for evidence of transcription of OmPLV genes and those in their flanking genomic regions. Two sets of *O. marina* RNA-seq transcriptomes were mapped to host contigs containing viral integrations. Most viral genes appear to be transcriptionally inactive (Figs 2a and S2), with only two cases showing moderate expression (contig_3421 and contig_15573, Fig. S2). Transcriptional activity was primarily concentrated in the gene-rich regions surrounding the viral insertions (Fig. S2). Interestingly, Ty1/copia LTR-retrotransposons were often located near the viral endogenizations (Figs 2 and S2), and in some cases, retrotransposon-associated genes show evidence of transcriptional activity (contig_10405 and contig_3421). The overall lack of transcriptional activity in most viral genes suggests that OmPLV is inactive; viral gene expression may be triggered by specific environmental conditions.

### OmPLV has a unique and diverse gene repertoire

OmPLV possesses a reduced virion morphogenesis module; genes for only two such proteins are found alongside unique replication proteins. We conducted a detailed annotation of OmPLV genes relative to the typical gene repertoire of polintons and related viruses [7,56]. TIRs ranging from 232 to 858 bp were identified in three OmPLV sequences (OmPLV_15573, OmPLV_3421 and OmPLV_9613) and served to delineate the boundaries of the endogenized viral genomes (Fig. 2d). Among the 12 protein-coding genes with discernible predicted functions, 3 were core proteins: MCP, A32_ATPase and pPolB. The morphogenesis module included only MCP and DNA-packaging ATPase (A32_ATPase). At the same time, genes for the minor capsid protein (mCP) and protease (PRO), typically present in most polintons, were absent or unrecognizable. Genes encoding endonuclease GIY-YIG and tyrosine recombinase (Y-rec) were identified and are likely involved in OmPLV integration, although the typical polinton integrase RVE-INT was not detected. Notably, OmPLV encodes a unique set of replication enzymes, including a novel fusion between pPolB and a helicase superfamily 1 (SF1H), forming the Helicase SF1–pPolB protein (see below), as well as an HJR, which is commonly found in large dsDNA viruses such as poxvirus and iridovirus [57].

A set of accessory genes with unclear functions in the context of viruses was also identified, including N6_Mtase, a collagen-like protein and a fused BRO-N–VRR-NUC gene. Notably, the BRO-N terminal domain was fused with the VRR-NUC endonuclease (Fig. S6C). The BRO-N domain is commonly seen in NCLDVs and phages [8,9], often fused to other functional domains [58]. However, the BRO-N–VRR-NUC fusion observed here in OmPLV has not been previously described. Additionally, evidence of gene degeneration was observed, including gene fragmentation into consecutive ORFs, likely due to frameshifts (e.g. the introduction of premature stop codons) (Fig. 2d). Also, long putative intergenic regions were detected within viral regions (Fig. 2d), which is consistent with ongoing endogenization and gene decay. It is unlikely that these regions are intronic insertions, as we did not find potential splice sites (GT-AG boundaries). Additionally, highly conserved pairs of TIRs were identified (230–860 nt, >90% identity) and intact Y-rec genes (>95% amino acid identity), whereas GIY-YIG integrase appears functionally impaired (Figs S7 and S8). Examination of the TIR-flanking regions detected no target-site duplications, consistent with Y-rec-mediated integration [59]. This apparent differential preservation of some genes suggests that at least some of these endogenized viral elements are not completely dead but are still capable of intragenomic mobilization (e.g. via Y-rec integration), supporting the idea of transitioning to a mobile element rather than simple decay after endogenization and silencing.

Given the low level of RNA-seq support for individual gene models, artefacts associated with imperfect ORF prediction cannot be ruled out. Despite this fragmentation, however, conserved gene arrangements were identified, such as the tail-to-tail orientation of MCP and Y-rec and the head-to-head configuration of BRO-N–VRR-NUC and ATPase (Figs 2d and S3), which may indicate functional associations and coordinated gene expression. All things considered, the chimeric gene content of OmPLV differs from the canonical gene content found in most polintons, PLVs and related viruses, suggesting that the OmPLV of *O. marina* is a novel type of PLV.

### OmPLV has a novel helicase-polymerase gene fusion

**Fig. 3.**
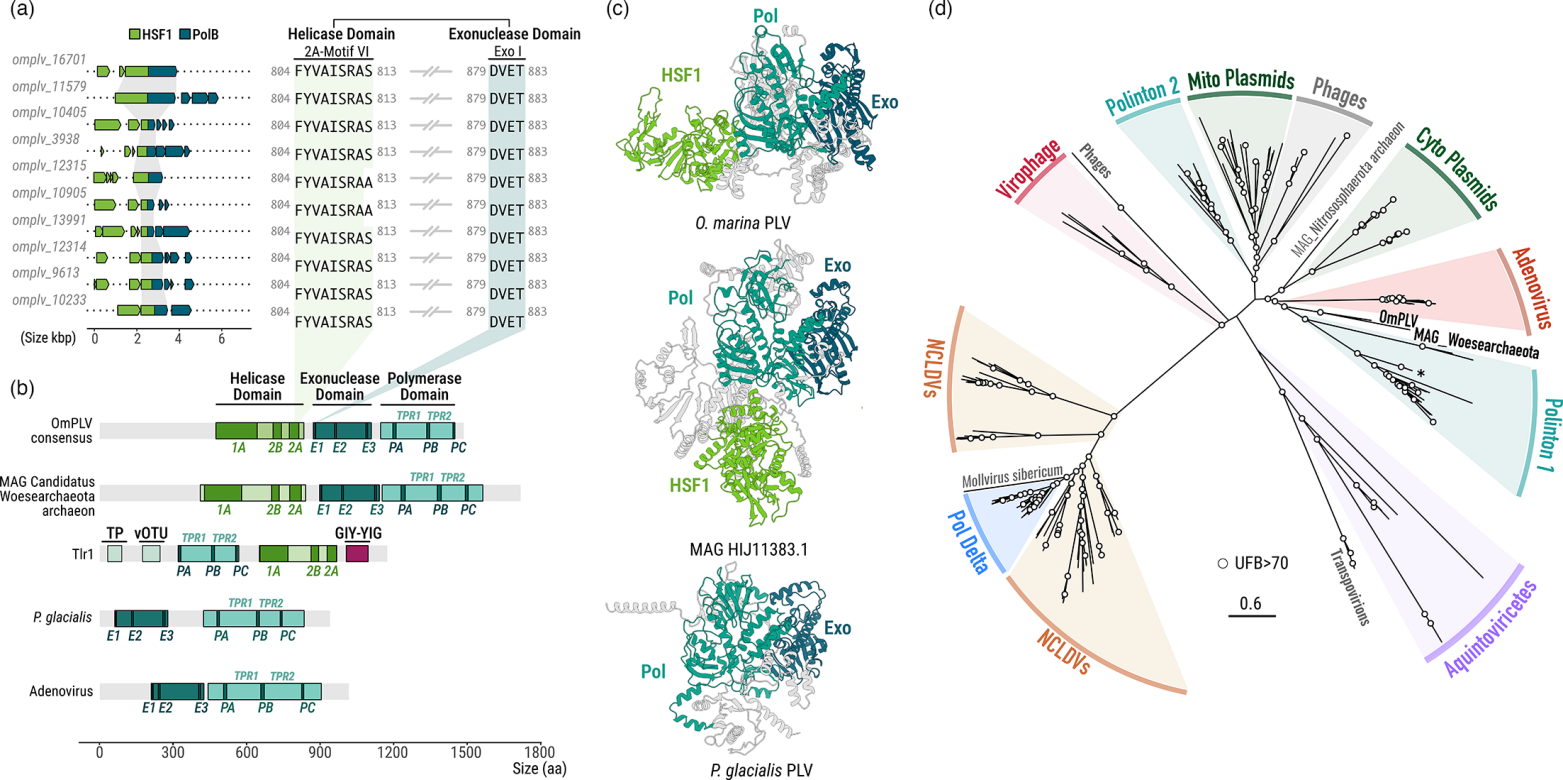
Physical structure and evolution of fused Helicase SF1–pPolB in *O. marina* OmPLV. (a) Gene fragmentation of the fused Helicase SF1–pPolB (left) and extraction of the protein region bridging Helicase (motif 2A-VI) and exonuclease (ExoI) domains (right) found in ten OmPLVs. Complete protein alignment is provided in Fig. S4. (b) Schematic representation and comparison of the protein domain organization of pPolB among adenovirus (O72539), the *P. glacialis* polinton (CCMP2088, contig scf7180000716369 [coordinates 74,982–77,801]), the fused Helicase SF1–pPolB of OmPLV, ‘*Candidatus Woesearchaeoata*’ MAG (HIJ11383) and the pPolB–Helicase SF1–GIY-YIG of *T. thermophila* Tlr1 element (AAL73455.1). Homologous domains are shown in similar colours, highlighting the conserved motifs of the Helicase SF1 (1A, 2B and 2A), exonuclease (E) and polymerase (P) domains. Overall pLDTT scores per structure: OmPLV, 68.36; MAG HIJ11383.1, 64.23; *P. glacialis* PLV, 86.41. (c) AlphaFold3 structure prediction for pPolB from ‘*Candidatus Woesearchaeoata*’ MAG, OmPLV and *P. glacialis* polinton. Exo, PolB and Helicase domains are coloured in dark blue, light blue and green, respectively. Predicted secondary structure alignments were conducted using ChimeraX v.1.8. (d) pPolB ML phylogenetic reconstruction (Q.pfam+F+R7, 374 amino acids). Branches are coloured according to their classification, including viral and non-viral elements such as a cytoplasmic linear plasmid (Cyto Plasmids), mitochondrial linear plasmid (Mito plasmids) and a subclass of eukaryotic pPolB (Delta). UFB support is indicated in white for nodes with >70% support. The scale bar shows inferred substitution per site.

Detailed comparative genomic investigation revealed that *O. marina* OmPLV possesses a highly distinctive gene encoding a large ~1,500 amino acid fused Helicase SF1–pPolB protein. Initial identification of the fusion gene was challenging due to its large size (~4.3 kbp) and the fact that most Helicase SF1–pPolB genes are fragmented in multiple (from three to seven) ORFs (Fig. 3a, left). However, some *in silico* translated protein fragments were long enough [e.g. OmPLV_11579_17 (944 amino acids) and OmPLV_16701_19 (806 amino acids)] to reveal the fusion between the two proteins and assist in identifying additional fusion genes. In total, the fusion was found in ten OmPLV elements (~96% amino acid identity in pairwise comparisons), bridging the conserved Helicase SF1 motif (motifs 2A–VI) and pPolB (Exo I) (Figs 3a, right, and S4). A complete 1,486 amino acid protein was identified, containing an N-terminal helicase with conserved domains 1A, 2B and 2A (sub-motifs V and VI) and a full pPolB with a complete exonuclease domain (motifs Exo1-3) and polymerase domain (motifs PolA-C) (Fig. 3b). Ongoing pseudogenization has likely contributed to the fragmentation of this otherwise persistent fusion.

Instances of fused polymerase genes have previously been reported in PLVs [6,7, 11], including Tlr1-PLV of the ciliate *T. thermophila* (Figs 1 and 3b) and transpovirions [11]. Notably, these fusions have an N-terminal polB and C-terminal Helicase, in contrast to the OmV SF1-pPolB architecture. Furthermore, the exonuclease domain is missing in all of these other cases (Fig. 3b). Intriguingly, a blastp search of NCBI databases using the novel Helicase SF1–pPolB as a query identified a gene for a 1,718 amino acid fusion variant (e-value: 2E-77, 26.1% identity) in a metagenomic assembly assigned as *Candidatus Woesearchaeota archaeon* [metagenomically assembled genome (MAG) HIJ11383.1], but more likely this contig represents a fragmented PLV genome. Together, the OmPLV and MAG HIJ11383.1 fusions represent the longest viral replication enzymes described thus far for *Preplasmiricota*, nearly twice the size of the pPolB found in dinoflagellate polintons (represented by *P. glacialis*) (Fig. 3b). Both Helicase SF1–pPolB variants described here (OmPLV and MAG HIJ11383.1) have long N-terminal regions of ~400 amino acids. No functional assignments could be made for either extension, such as TP and vOTU, that have been found on other polymerase fusions [4]. Although two discrete domains were predicted for both N-terminal regions using AlphaFold, confidence scores were low (Fig. S5). Secondary structure analysis of the fused Helicase SF1–pPolB nevertheless revealed high similarity between the OmPLV and MAG HIJ11383.1 N-terminal variants. It also confirmed the presence of TPR1 and TPR2 subdomains within the palm polymerase domain, supporting their classification as members of the protein-primed DNA polymerase family B (Fig. S5).

To elucidate the evolutionary history of the OmPLV helicase-polymerase gene fusion, we performed a broader phylogenetic analysis of the pPolB domain, including homologues from similar viruses and non-viral elements, following the strategy of [6,56]. The analysis revealed similar branching patterns to those seen in these studies, with the pPolB sequences associated with mitochondrial plasmids (Mito plasmids) and Polinton group 2 (Polinton2) branching with phages (Fig. 3d). The pPolBs of OmPLV and MAG HIJ11383.1 branch robustly together and between adenovirus and Polinton group 1 homologues, including those identified in dinoflagellates (Fig. 3d), suggesting that OmPLV and MAG HIJ11383.1 pPolB share a common virus-like ancestor. Consistent with the observed differences in domain organization, the phylogeny also indicates that the Helicase SF1–pPolB fusion in OmPLV and MAG HIJ11383.1 originated independently from the other fusion proteins found in Aquintoviricetes (e.g. Tlr and *Bigelowiella natans* PLV) and related non-viral elements (transpovirions) (Fig. 3d).

### OmPLV is evolutionarily distinct

**Fig. 4.**
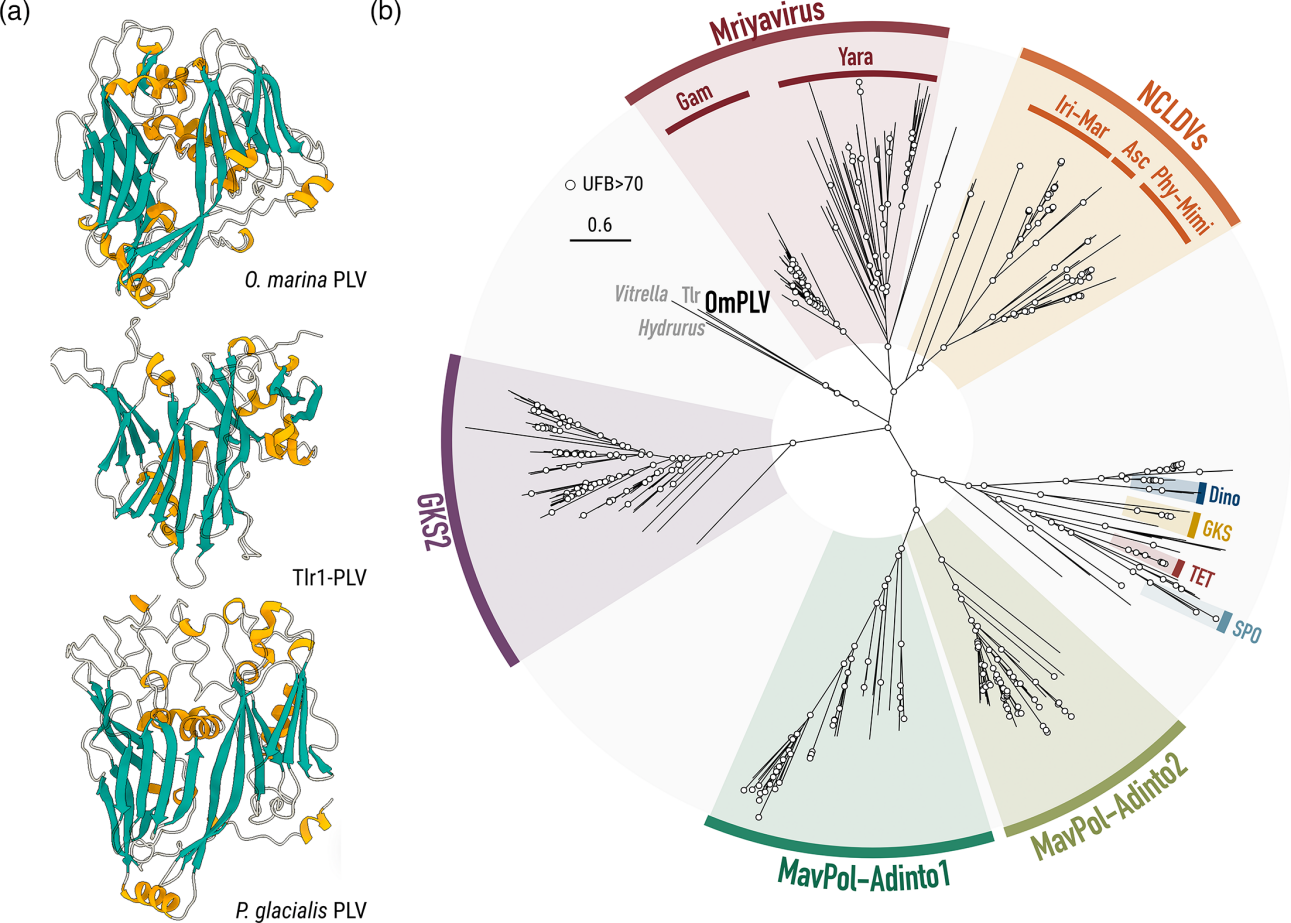
Structure prediction and evolution of MCP of OmPLV. (a) Secondary structure prediction of the DJR MCP for OmPLV, *T. thermophila* Tlr1 viral element and *P. glacialis* polinton. Beta-strands, alpha helices and coil domains are highlighted in green, yellow and white, respectively. Overall pLDTT score per structure: OmPLV, 73.17; Tlr1-PLV, 34.35; *P. glacialis* PLV, 84.28. (b) ML phylogeny (LG+G+F model, 451 amino acids) of the MCP across representative groups of polintons, PLVs, Mriyavirus and NCLDVs. Distinctive colours represent different groups described in previous studies [6,7, 18, 19, 42, 67]. Abbreviations: Gam, Gamadvirus; Yara, Yaravirus; Iri, Iridovirus; Mar, Marseillevirus; Asc, Ascovirus; Phy, Phycovirus; Mimi, Mimivirus; Dino, Dinoflagellate PLVs; GKS, Gossenköllesee lake PLVs; SPO, South Pacific Ocean PLVs; TET, Tetraselmis PLVs; MavPol-Adinto, maverick/polinton and adintovirus. The phylogenetic position of OmPLV is highlighted in black, along with closely related viral elements. UFB support is indicated in white for nodes with >70% support. The scale bar depicts inferred substitution per site.

Secondary structure prediction and phylogenetic analysis of MCPs were used to investigate the evolutionary relationship of OmPLV with related viruses, including polintons, PLVs, NCLDVs and the recently described Mriyavirus [6,7, 18, 19, 42]. The OmPLV MCP (Fig. 4a) has the DJR structure – two tandem eight *ß*-strands, each composed of two *ß*-sheets – characteristic of the MCPs of dsDNA viruses within the kingdom Bamfordvirae [4,8, 60].

The MCP phylogenetic tree shows that OmPLV is distinct from previously characterized groups of small dsDNA viruses (Fig. 4b). Unexpectedly, OmPLV does not branch with previously reported dinoflagellate polintons (‘Dino’ in Fig. 4b); instead, it forms a clade with divergent, previously unplaced, PLVs (i.e. Tlr1 and the elements from the alveolate *Vitrella* and the chrysophycean *Hydrurus*). This newly defined lineage occupies a phylogenetic position between established PLV groups and NCLDVs. However, the exact evolutionary relationships remain uncertain due to high sequence divergence and limited MCP sequences of related viruses. The phylogenetic affinity between OmPLV and NCLDVs is more evident in the HJR and ATPase trees, where OmPLV groups closely with homologues from poxviruses and Mininucleoviridae, respectively (Figs S6 and S7). Furthermore, MCP phylogeny confirms well-defined monophyletic clusters for established PLV groups (SPO, Tetra-PLV, GKS and GKS2) and *Eupolintoviridae* (adintovirus groups 1 and 2) (Fig. 4b). Overall, OmPLV appears to represent a new lineage of PLV that includes divergent PLVs such as the Tlr1 element in the ciliate *T. thermophila*.

## Discussion

We have discovered a new endogenous small dsDNA virus that is a prominent feature of the genome of the dinoflagellate *O. marina*. Dinoflagellates are among the most abundant marine protists and are noteworthy for their exceptionally large nuclear genomes. Only a few instances of dsDNA endogenized viruses have thus far been documented in the group [18,61]. Despite the ultra-large size of the *O. marina* genome (30–50 Gbp [62]), our limited genomic assembly contained many endogenized copies of OmPLV. This underscores the effectiveness of long-read sequencing in helping to identify and contextualize viral regions in even the largest and most complex nuclear genomes. These findings align with recent research suggesting that endogenized viral elements are more common and biologically significant in protists and algae than previously thought [18,63, 64]. Indeed, evidence for past associations between large DNA viruses and dinoflagellates comes from the fact that in all examined species, histone proteins have been replaced by dinoflagellate/viral nuclear proteins (DVNPs) as the main basic DNA-associated protein [61,65]. The precise functions of DVNPs are still unclear, but they appear to be an essential and critical component of a unique chromatin organization. The most plausible evolutionary scenario is that DVNPs were acquired by an ancestor of extant dinoflagellates from an NCLDV of the general type that today infects photosynthetic stramenopiles, such as giant kelp (*Macrocystis pyrifera*) or pelagophytes (*Aureococcus anophagefferens*), which are known hosts for large DNA viruses (e.g. *Phycodnaviridae*). The existence of DVNPs thus constitutes a prime example of the long-term impact of viral endogenization on the genome biology and evolution of eukaryotes [66].

### OmPLV and the transition to a TE lifestyle

Comparative genomic investigation of the 28 distinct OmPLVs identified herein suggests that this virus is adopting a TE lifestyle. Although some gene clusters are still conserved between the different *O. marina* copies (e.g. MCP and Y-rec; ATPase and BRO-N–VRR-NUC), extensive gene fragmentation and the presence of non-coding regions indicate ongoing viral genome disruption. Additionally, the consistent and uniform endogenization signatures (i.e. low GC content and codon usage bias) and the absence of significant MCP sequence variation between the genomic variants suggest that OmPLV originated from one or a limited number of infection and endogenization events. Interestingly, OmPLV appears to lack certain proteins essential for virion assembly, such as mCP and protease, which are commonly present in other Preplasmiviricota viruses [8] but absent in some PLVs [43]. OmPLV may thus employ an alternative virion assembly mechanism using homologous proteins or may not need them at all. In any case, endogenization does not preclude replication and movement as a TE. The presence of retrotransposons close to OmPLV in the *O. marina* genome (Fig. S2) seems important; this association has been noticed in the heterotrophic stramenopile *Cafeteria burkhardae* and several other protists [18,35]. It remains to be determined whether retrotransposons participate in the mobilization of proviral elements or hijack reactivated viruses. A dual lifestyle is also possible, as suspected for PLVs in the green alga *Tetraselmis* [67] and as observed in Mu-like bacteriophages that acquired transposition capabilities while remaining bona fide viruses [68]. There is still much to learn about the transition from bona fide viruses to mobile genetic elements.

### Chimeric gene repertoire of OmPLV

OmPLV is remarkably heterogeneous. Each copy identified in the *O. marina* genome has a rare gene arrangement and a distinct chimeric gene composition not found in other viruses, including genes typically associated with NCLDVs and phages. OmPLV resembles Aquintoviricetes and some virophages in encoding a tyrosine recombinase (Y-rec) [7,69]. However, the precise mechanism by which PLVs are integrated remains unknown. GIY-YIG endonuclease is also encoded and may be associated with viral homing [5]. Additionally, OmPLV encodes an HJR-like protein, likely derived from NCLDVs, which may play a role in resolving complex recombinant structures formed during viral integration. The presence of genes associated with integration and recombination in OmPLV speaks to its potential for mobility and ability to adapt to diverse genomic contexts.

OmVLP has a gene encoding a VRR-NUC endonuclease domain fused with a DNA-binding BRO-N domain, a combination not previously described in polintons or related viruses. The BRO-N domain is derived from the BRO protein described in *Baculoviridae* (i.e. Baculovirus Repeated ORF) and is known for its high affinity for DNA [58]. Several functions have been proposed for this domain, mostly related to regulating phage transcription and replication [70,71]. On the other hand, the VRR-NUC domain is known to bind and cleave specific DNA structures [72], similar to the TALEN genome-editing system[73]. This novel fusion protein in OmVLP may thus play a role in viral dynamics, perhaps involved in viral genome integration or excision. However, the functional role of these domains in PLVs has yet to be determined. All things considered, the unique gene repertoire of OmPLV underscores its unique and divergent nature. Further exploration of non-core genes will be crucial to understanding essential viral processes such as induction.

### Evolutionary significance of OmPLV replication fusion protein

OmPLV is noteworthy in its possession of a gene encoding a ~1,500 amino acid fused Helicase SF1–pPolB protein, a fusion that was found to have a ~1,800 amino acid homologue in a marine MAG designated ‘*Candidatus Woesearchaeota*’. Together, these fusion proteins represent the largest viral replication enzymes known for Preplasmiviricota. A few pPolB-helicase fusions have been described in PLVs, e.g. the *T. thermophila* Tlr-helicase, which contains only the polymerase domain followed by a C-terminal Helicase SF1 domain [4,11]. In contrast, the OmPLV Helicase SF1–pPolB discovered herein (and its counterpart in a prokaryotic MAG; below) retains the full pPolB (i.e. exonuclease and polymerase domains), with the helicase domain located at the N-terminal region. Recently, two N-terminal domains (TP and vOTU) were identified in Tlr-helicases, likely linked to protein priming replication and polyprotein cleavage [4]. However, these domains were not found in OmPLV, suggesting it represents a unique viral replication enzyme.

pPolB is a key protein for understanding the evolution of dsDNA viruses within Bamfordvirae [8]. Published pPolB phylogenetic analyses differentiate between eukaryotic and prokaryotic viruses and position polintons as a basal group from which mitochondrial plasmids, cytoplasmic plasmids, virophages and adenoviruses are derived [8,11, 12]. This evolutionary scenario has been supported by the discovery of the pPolBs with N-terminal domains discussed herein [3,4]. The replacement of the pPolB exonuclease domain by a Helicase SF1 domain likely occurred in an ancestral polinton, leading to the emergence of a Tlr-helicase-like entity [3,4]. Interestingly, while the distinct domain organizations of the OmPLV SF1-pPolB and Tlr fusions suggest independent origins, they likely evolved in related lineages, i.e. dinoflagellates and ciliates, respectively (both of which are alveolates). Indeed, in MCP phylogenetic trees (Fig. 4b), OmPLV and Tlr branch together as part of a distinct, divergent lineage. Furthermore, phylogenetic trees of PolB reveal that these domains in OmPLV and Tlr are not orthologous (Tlr1 lacks the exonuclease domain). Taken together, these findings suggest that although the OmPLV and Tlr lineages are related in a broad sense, they underwent gene innovation and exchange independent of one another.

Regarding the OmPLV homologue in ‘*Candidatus Woesearchaeota*’, attempts to extract additional information from the ~9,300 bp MAG were unsuccessful; no other genes with obvious homology to known proteins could be detected. The identity of this MAG sequence and its relationship to OmPLV remain a mystery, but it hints at the existence of hitherto undescribed viral diversity in archaeal tectiviruses. It is conceivable that OmPLV stems from an ancestral lineage that predates the diversification of present-day polintons and viruses. Alternatively, the fused polymerase may have evolved independently in OmPLV and tectiviruses, suggesting convergent evolution rather than shared ancestry with polintons. The origin of polinton and PLV diversity may not be as streamlined and may involve multiple events of gene fusion and replacement.

## Conclusion

We have identified a novel ~25 kbp dsDNA virus related to Preplasmiviricota and closely associated with NCLDVs. OmPLV is endogenized within the *O. marina* genome and shows evidence of a TE-like lifestyle. OmPLV features a mosaic gene content and a unique fused Helicase SF1–pPolB replication enzyme. Such gene fusions constitute diagnostic features that can help elucidate the diversity and evolutionary history of dsDNA viruses. Our results demonstrate that genomic surveys based on long-read sequencing can effectively resolve and assemble complex viral elements and detect their integration into large genomes such as those of dinoflagellates, which are highly plastic and include a significant fraction of active TEs. This may facilitate viral integration and propagation. Dinoflagellates are highly prevalent in aquatic ecosystems; our study serves as a foundation for further sequencing efforts in less-studied dinoflagellates to uncover new viral elements and help us understand the impact of viruses on eukaryotic genome biology and evolution.

## Supplementary material

10.1099/jgv.0.002200 Uncited Supplementary Material 1.

## Abbreviations

DJR: double jelly-roll
DVNPs: dinoflagellate/viral nuclear proteins
HJR: Holliday junction resolvase
MAG: metagenomically assembled genome
MCP: major capsid protein
ML: maximum likelihood
NCBI: National Center for Biotechnology Information
NCLDVs: *Nucleocytoviricota* viruses
OmPLV: *Oxyrrhis marina* PLV
PLV: polinton-like virus
SCUO: synonymous codon usage orderliness
TEs: transposable elements
TIRs: terminal inverted repeats
TP: terminal protein
vOTU: viral ovarian tumour
