# Supplementary material for: Endogenized polinton-like viruses in the dinoflagellate Oxyrrhis marina uncover novel PolB fusion
Source: J Gen Virol. 2025 Dec 22;106(12):002200. doi: 10.1099/jgv.0.002200 (PMC12721341; doi:10.1099/jgv.0.002200)
Supplement: Uncited Supplementary Material 1. [file jgv-106-02200-s001.pdf]

SUPPLEMENTARY INFORMATION

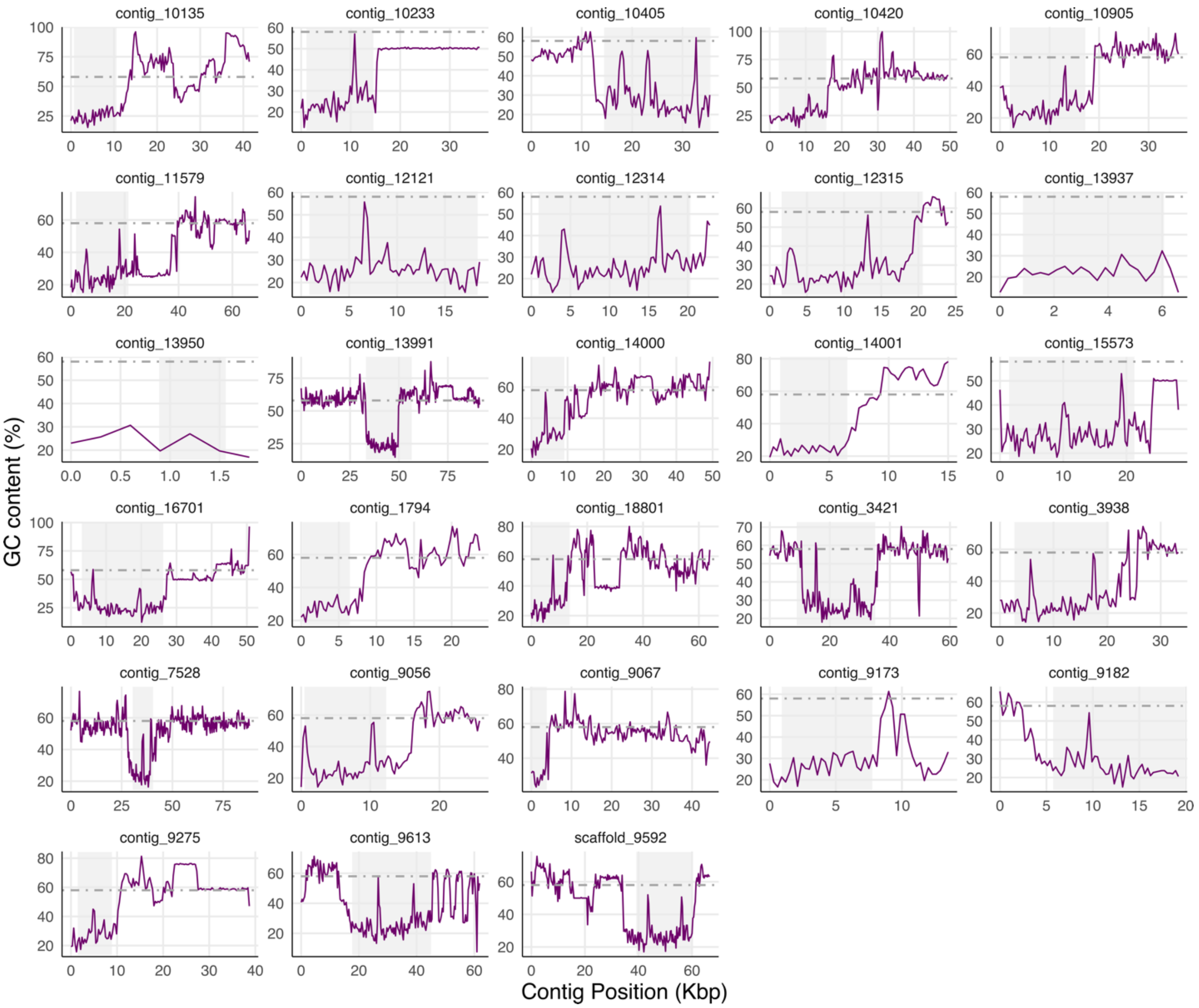

Fig. S1. GC content deviation of OmPLV in genomic host contigs. GC content distribution: Purple skew represents GC content fluctuation along the contig, and the viral regions are highlighted in light gray.

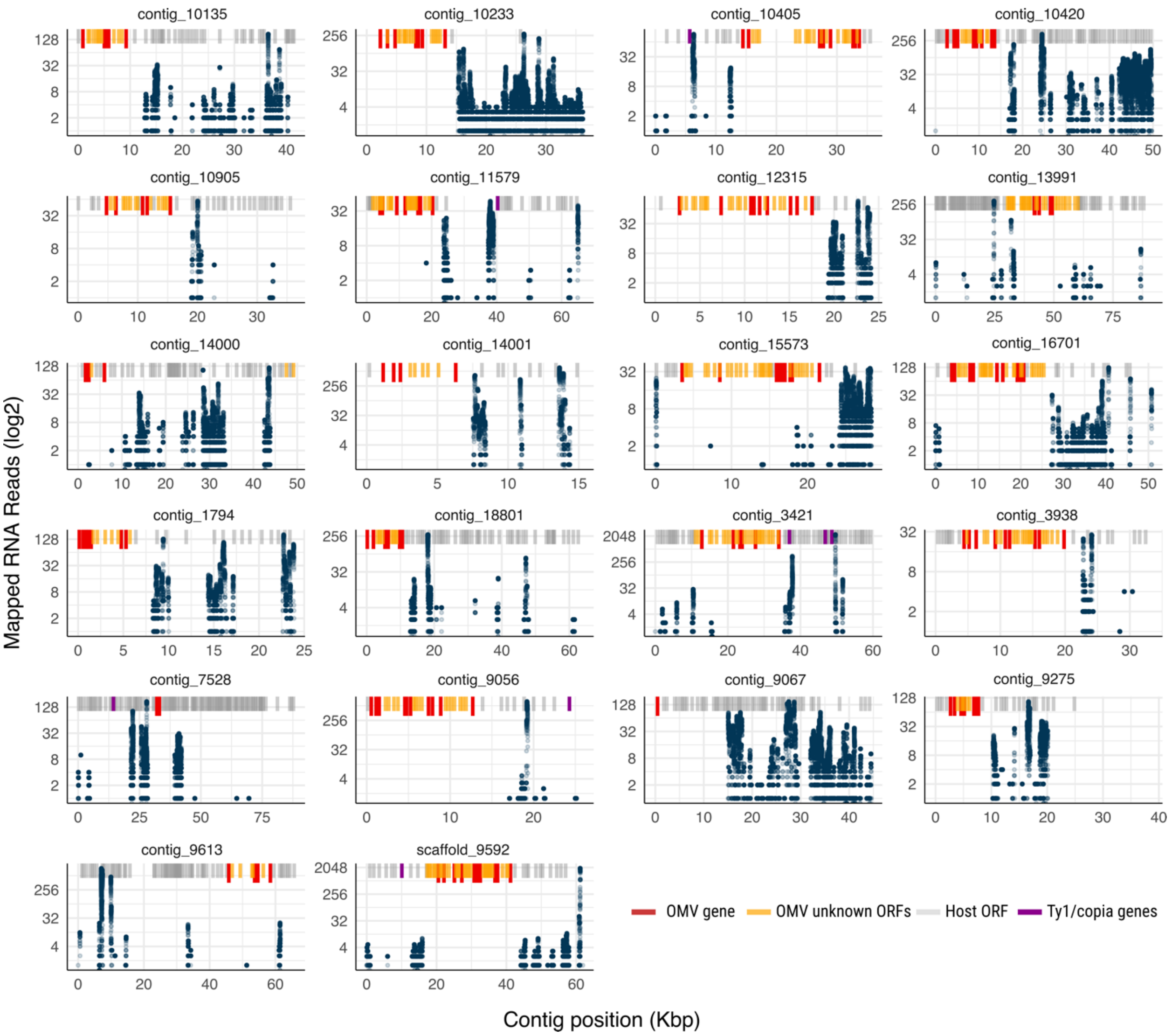

Fig. S2. RNA-seq reads mapping patterns on the host contigs. Blue dots are the number of RNA-seq reads (y-axis) mapped to a particular position of the *O. marina* contigs (x-axis). Red and orange genes depict integrated viral regions. The purple ribbon shows the location of Ty1/copia LTR-retrotransposon.

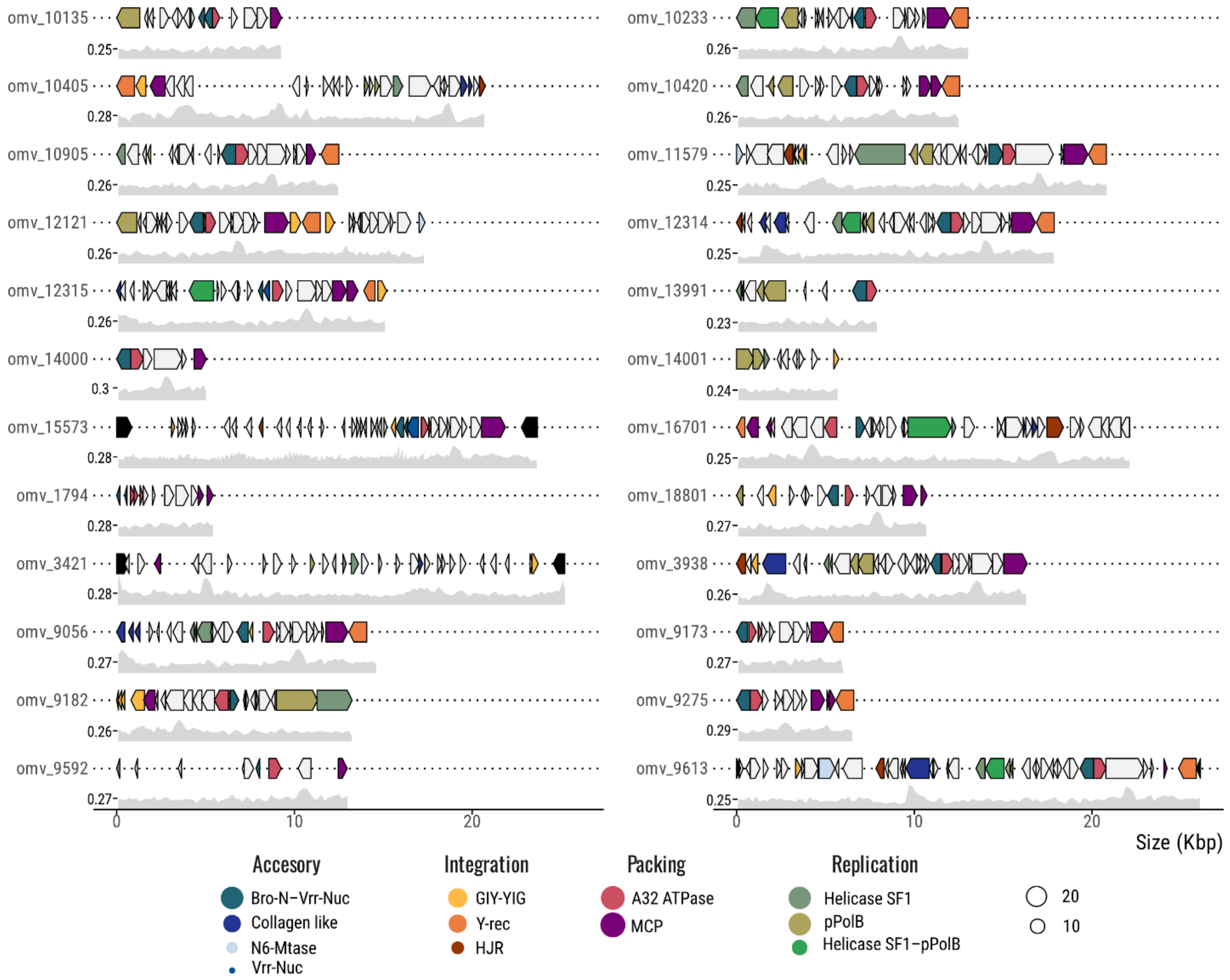

Fig. S3. Partial and complete genomes of 24 OmPLV were identified from the sequencing dataset. The remaining four OmPLVs were reduced to a couple of genes (less than < 5kbp). Genes are coloured according to the legend, and the GC content is depicted below each genome. In the legend, the size of the circle represents the gene frequency of OmPLV.

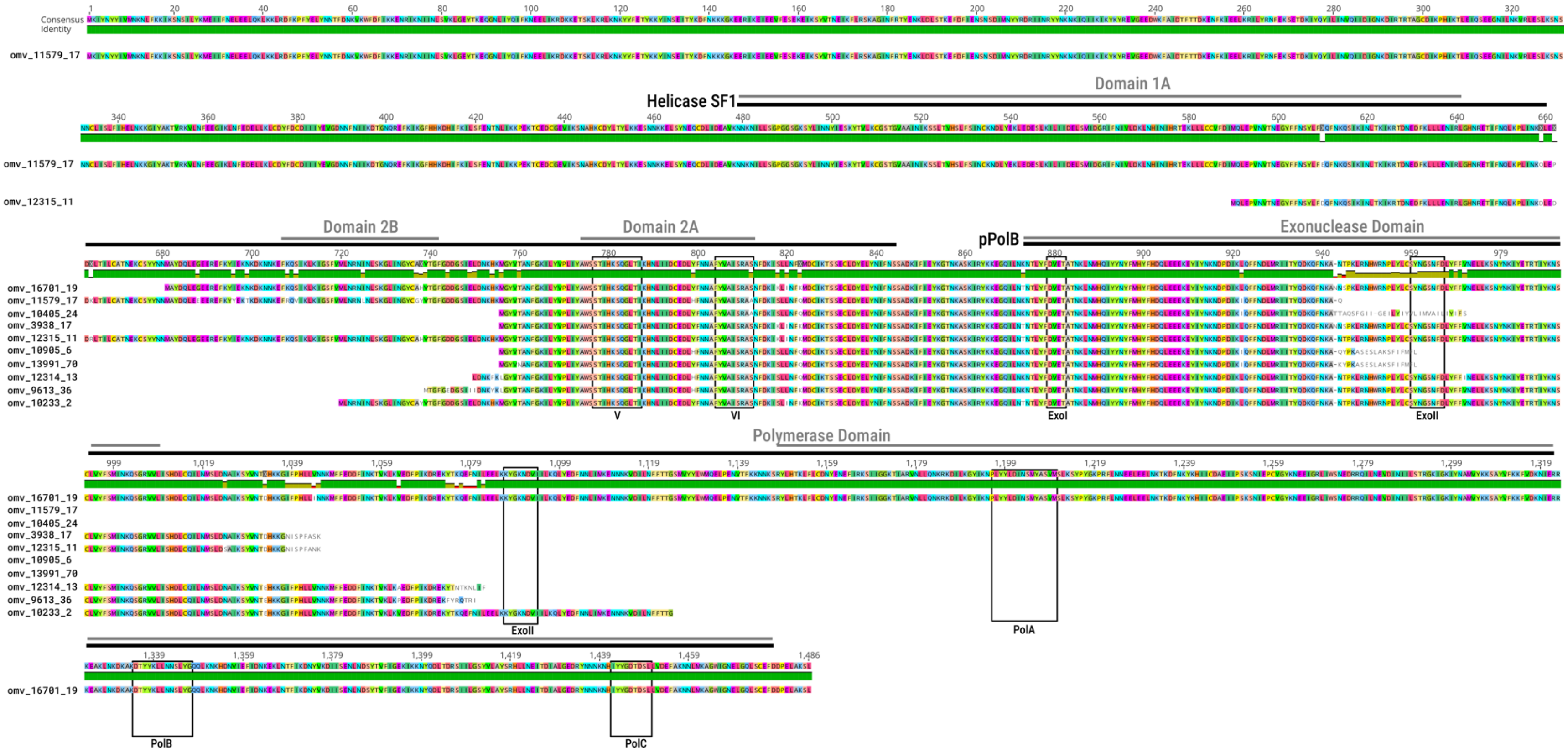

Fig. S4. Fused Helicase SF1-pPolB amino acid reconstruction. Amino acid alignment of the peptides that include the fused protein region.

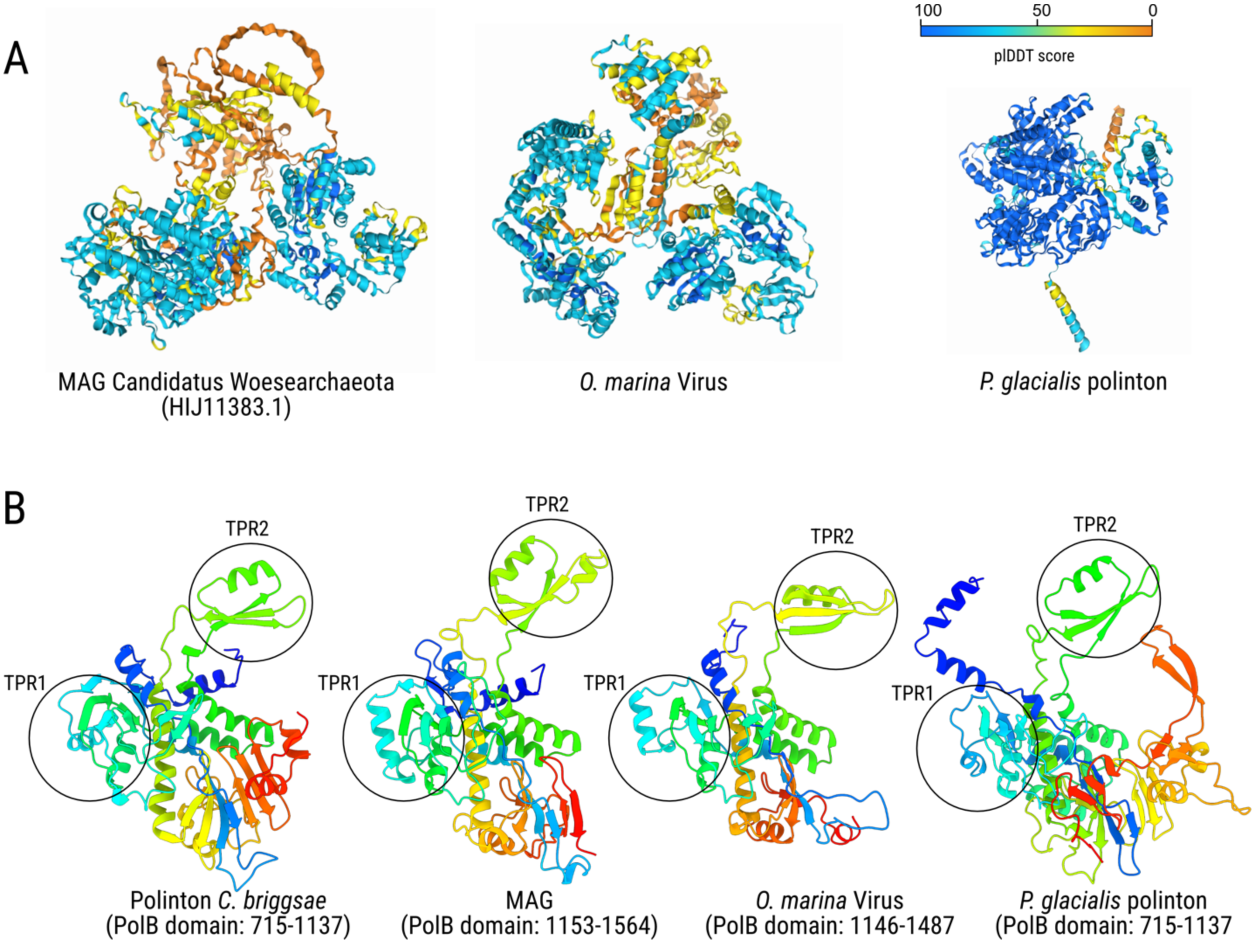

Fig. S5. Secondary structure prediction for pPolB and identification of TPR1 and TPR2 subdomains. A. structural model of pPolB coloured according to their confidence score: predicted local distance difference test (plDDT) obtained from AlphaFold3. **B**. identification of the specific TPR1 and TPR2 subdomain confirming the pPolB identity.

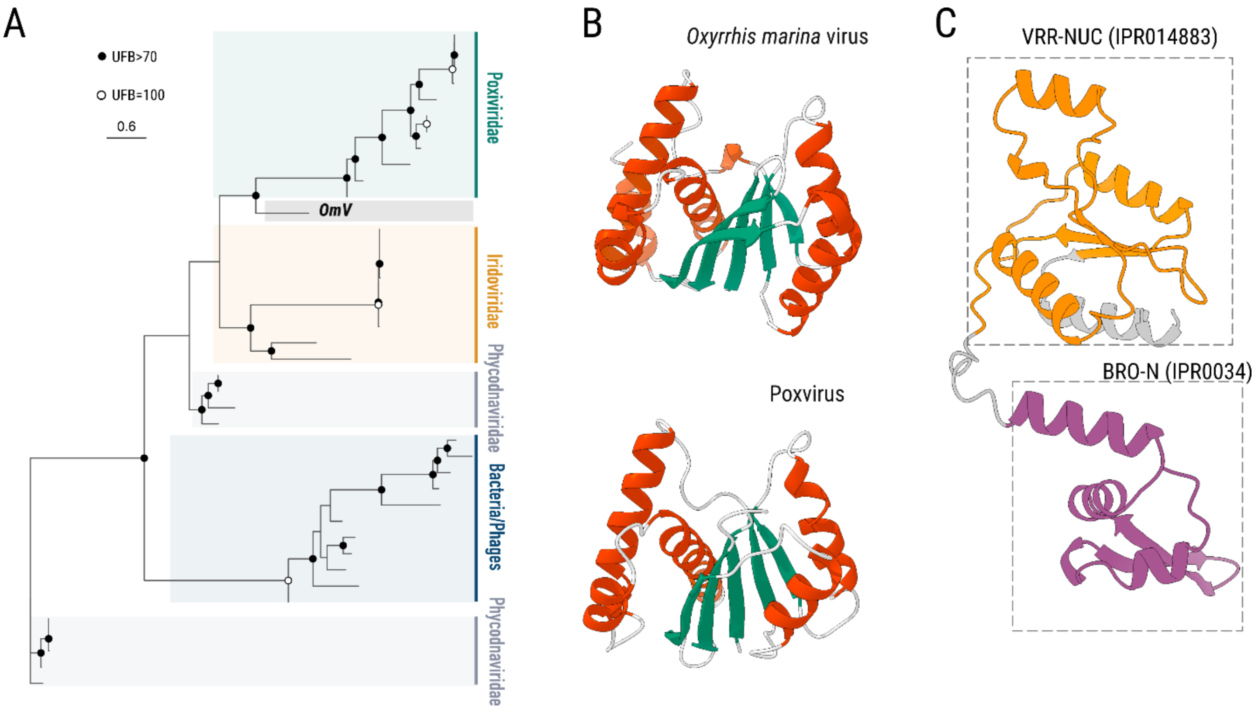

Fig. S6. Secondary structure prediction for HJR and fused BRO-N –VRR-NUC domains and phylogeny of HJR. A. Maximum likelihood phylogeny of HJR, including virus in which this protein is frequently found (Model and aa length). AplphaFold2 secondary structure prediction for HJR (**B**) and a novel fusion for BRO-N domain with VRR-NUC (**C**).

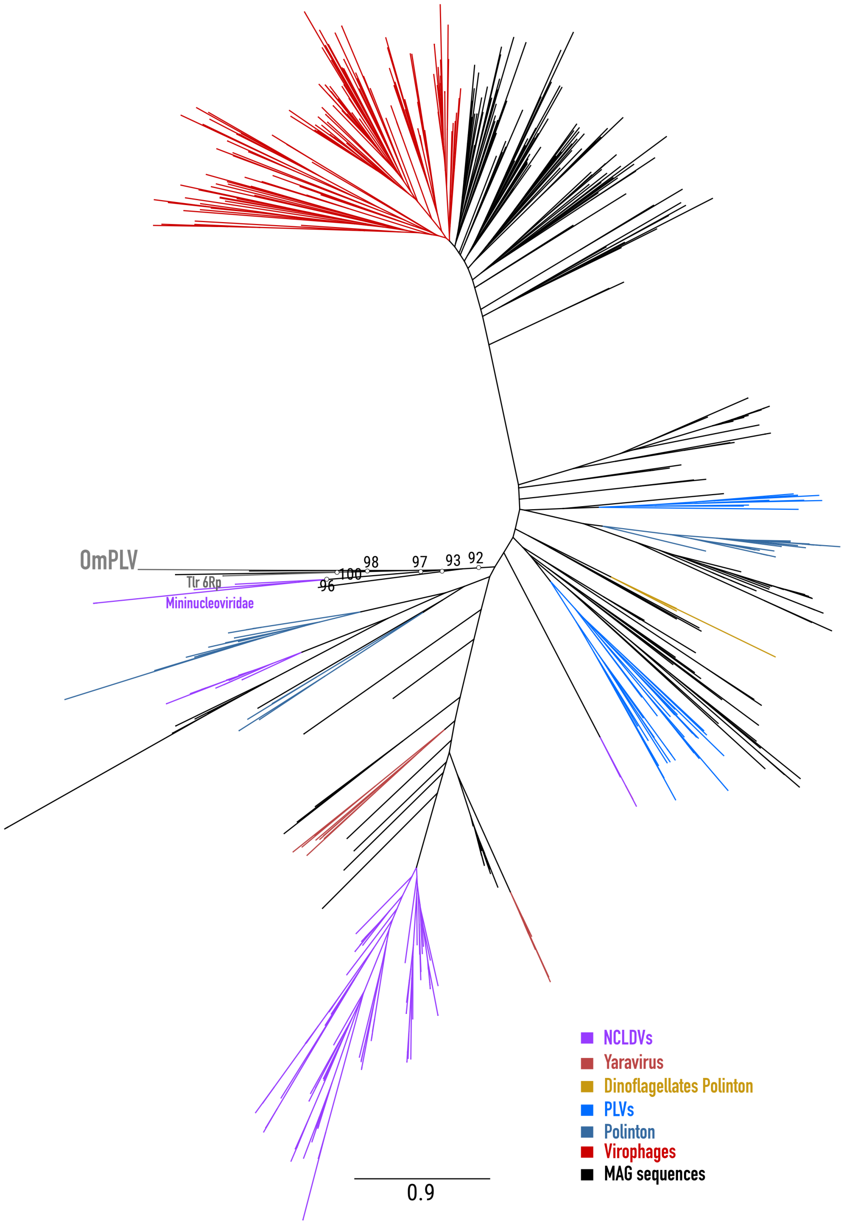

Fig. S7. ATPase phylogeny. Maximum likelihood phylogeny (Q.pfam+F+R8 and 1,000 ufb replicates), including major groups of dsDNA viruses depicted according to the legend. OmPLV clusters with Tlr PLV and branches alongside Mininucleoviridae (1). Bootstrap support is shown only for the branches related to OmPLV, and full details about the tree topology and bootstrap support are available in the supplementary information.

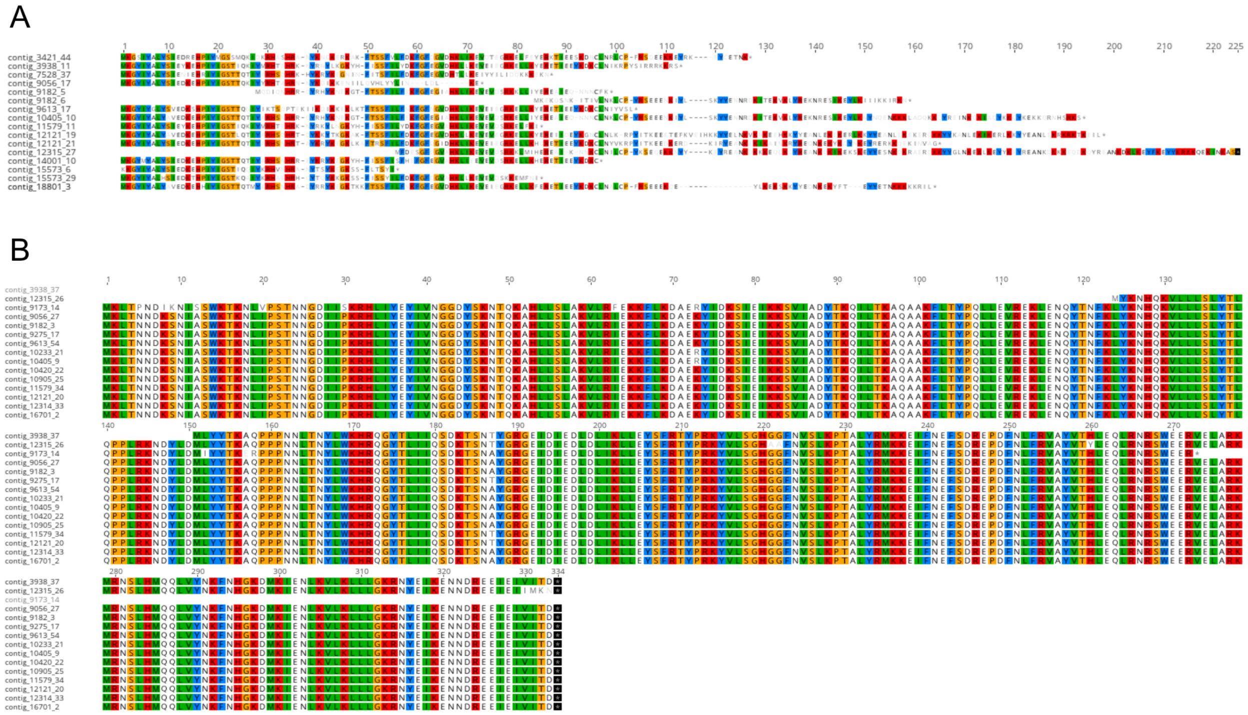

Fig. S8. Amino acid sequence conservation GIY-YIG (A) and Y-rec (B).
